# Supplementary material for: Pyruvate Dehydrogenase Kinase 1 inhibition mediated oxidative phosphorylation enhancement in cartilage promotes osteoarthritis progression
Source: BMC Musculoskelet Disord. 2023 Jul 20;24:597. doi: 10.1186/s12891-023-06585-6 (PMC10357736; doi:10.1186/s12891-023-06585-6)
Supplement: Supplementary file 1 — Additional file 1. [file 12891_2023_6585_MOESM1_ESM.zip › KEGG.Gsea.1653622432464/KEGG_TYPE_II_DIABETES_MELLITUS.html]

Details for gene set KEGG\_TYPE\_II\_DIABETES\_MELLITUS[GSEA]

|  || Dataset | 55457\_collapsed\_to\_symbols.cls.cls #OA\_versus\_normal.cls.cls #OA\_versus\_normal\_repos |
| Phenotype | cls.cls#OA\_versus\_normal\_repos |
| Upregulated in class | normal |
| GeneSet | KEGG\_TYPE\_II\_DIABETES\_MELLITUS |
| Enrichment Score (ES) | -0.34051675 |
| Normalized Enrichment Score (NES) | -1.2525522 |
| Nominal p-value | 0.13527398 |
| FDR q-value | 0.31150794 |
| FWER p-Value | 1.0 |
Table: GSEA Results Summary

  

Fig 1: Enrichment plot: KEGG\_TYPE\_II\_DIABETES\_MELLITUS      
 Profile of the Running ES Score & Positions of GeneSet Members on the Rank Ordered List

  

| SYMBOL | TITLE | RANK IN GENE LIST | RANK METRIC SCORE | RUNNING ES | CORE ENRICHMENT || 1 | MAPK9 | mitogen-activated protein kinase 9 [Source:HGNC Symbol;Acc:HGNC:6886] | 721 | 0.588 | -0.0117 | No |
| 2 | MAPK3 | mitogen-activated protein kinase 3 [Source:HGNC Symbol;Acc:HGNC:6877] | 757 | 0.579 | 0.0292 | No |
| 3 | TNF | tumor necrosis factor [Source:HGNC Symbol;Acc:HGNC:11892] | 2098 | 0.350 | -0.0487 | No |
| 4 | PIK3CB | "phosphatidylinositol-4,5-bisphosphate 3-kinase catalytic subunit beta [Source:HGNC Symbol;Acc:HGNC:8976]" | 2186 | 0.340 | -0.0298 | No |
| 5 | PRKCD | protein kinase C delta [Source:HGNC Symbol;Acc:HGNC:9399] | 2237 | 0.335 | -0.0085 | No |
| 6 | PRKCZ | protein kinase C zeta [Source:HGNC Symbol;Acc:HGNC:9412] | 2760 | 0.274 | -0.0284 | No |
| 7 | ABCC8 | ATP binding cassette subfamily C member 8 [Source:HGNC Symbol;Acc:HGNC:59] | 2844 | 0.266 | -0.0148 | No |
| 8 | SLC2A2 | solute carrier family 2 member 2 [Source:HGNC Symbol;Acc:HGNC:11006] | 3168 | 0.234 | -0.0223 | No |
| 9 | HK3 | hexokinase 3 [Source:HGNC Symbol;Acc:HGNC:4925] | 3792 | 0.178 | -0.0574 | No |
| 10 | INS | insulin [Source:HGNC Symbol;Acc:HGNC:6081] | 3885 | 0.166 | -0.0520 | No |
| 11 | PKM | pyruvate kinase M1/2 [Source:HGNC Symbol;Acc:HGNC:9021] | 3963 | 0.159 | -0.0460 | No |
| 12 | CACNA1G | calcium voltage-gated channel subunit alpha1 G [Source:HGNC Symbol;Acc:HGNC:1394] | 4422 | 0.119 | -0.0727 | No |
| 13 | IKBKB | inhibitor of nuclear factor kappa B kinase subunit beta [Source:HGNC Symbol;Acc:HGNC:5960] | 5914 | 0.001 | -0.1885 | No |
| 14 | PRKCE | protein kinase C epsilon [Source:HGNC Symbol;Acc:HGNC:9401] | 5926 | 0.000 | -0.1894 | No |
| 15 | IRS4 | insulin receptor substrate 4 [Source:HGNC Symbol;Acc:HGNC:6128] | 6106 | -0.013 | -0.2023 | No |
| 16 | CACNA1C | calcium voltage-gated channel subunit alpha1 C [Source:HGNC Symbol;Acc:HGNC:1390] | 6367 | -0.033 | -0.2201 | No |
| 17 | HK1 | hexokinase 1 [Source:HGNC Symbol;Acc:HGNC:4922] | 6728 | -0.058 | -0.2437 | No |
| 18 | MAPK1 | mitogen-activated protein kinase 1 [Source:HGNC Symbol;Acc:HGNC:6871] | 6853 | -0.068 | -0.2483 | No |
| 19 | PIK3CD | "phosphatidylinositol-4,5-bisphosphate 3-kinase catalytic subunit delta [Source:HGNC Symbol;Acc:HGNC:8977]" | 7405 | -0.111 | -0.2827 | No |
| 20 | CACNA1E | calcium voltage-gated channel subunit alpha1 E [Source:HGNC Symbol;Acc:HGNC:1392] | 7540 | -0.121 | -0.2840 | No |
| 21 | PIK3CA | "phosphatidylinositol-4,5-bisphosphate 3-kinase catalytic subunit alpha [Source:HGNC Symbol;Acc:HGNC:8975]" | 7733 | -0.136 | -0.2887 | No |
| 22 | CACNA1A | calcium voltage-gated channel subunit alpha1 A [Source:HGNC Symbol;Acc:HGNC:1388] | 7774 | -0.140 | -0.2813 | No |
| 23 | ADIPOQ | "adiponectin, C1Q and collagen domain containing [Source:HGNC Symbol;Acc:HGNC:13633]" | 7792 | -0.141 | -0.2720 | No |
| 24 | PIK3CG | "phosphatidylinositol-4,5-bisphosphate 3-kinase catalytic subunit gamma [Source:HGNC Symbol;Acc:HGNC:8978]" | 7953 | -0.152 | -0.2730 | No |
| 25 | PIK3R3 | phosphoinositide-3-kinase regulatory subunit 3 [Source:HGNC Symbol;Acc:HGNC:8981] | 8801 | -0.217 | -0.3226 | Yes |
| 26 | PIK3R5 | phosphoinositide-3-kinase regulatory subunit 5 [Source:HGNC Symbol;Acc:HGNC:30035] | 8804 | -0.217 | -0.3064 | Yes |
| 27 | MAPK8 | mitogen-activated protein kinase 8 [Source:HGNC Symbol;Acc:HGNC:6881] | 8956 | -0.228 | -0.3009 | Yes |
| 28 | CACNA1B | calcium voltage-gated channel subunit alpha1 B [Source:HGNC Symbol;Acc:HGNC:1389] | 9092 | -0.239 | -0.2934 | Yes |
| 29 | MAPK10 | mitogen-activated protein kinase 10 [Source:HGNC Symbol;Acc:HGNC:6872] | 9699 | -0.291 | -0.3186 | Yes |
| 30 | SOCS2 | suppressor of cytokine signaling 2 [Source:HGNC Symbol;Acc:HGNC:19382] | 9802 | -0.302 | -0.3038 | Yes |
| 31 | CACNA1D | calcium voltage-gated channel subunit alpha1 D [Source:HGNC Symbol;Acc:HGNC:1391] | 9867 | -0.308 | -0.2855 | Yes |
| 32 | HK2 | hexokinase 2 [Source:HGNC Symbol;Acc:HGNC:4923] | 10030 | -0.325 | -0.2736 | Yes |
| 33 | PKLR | pyruvate kinase L/R [Source:HGNC Symbol;Acc:HGNC:9020] | 10120 | -0.333 | -0.2554 | Yes |
| 34 | GCK | glucokinase [Source:HGNC Symbol;Acc:HGNC:4195] | 10137 | -0.335 | -0.2314 | Yes |
| 35 | PDX1 | pancreatic and duodenal homeobox 1 [Source:HGNC Symbol;Acc:HGNC:6107] | 10409 | -0.362 | -0.2253 | Yes |
| 36 | SLC2A4 | solute carrier family 2 member 4 [Source:HGNC Symbol;Acc:HGNC:11009] | 10764 | -0.400 | -0.2226 | Yes |
| 37 | PIK3R2 | phosphoinositide-3-kinase regulatory subunit 2 [Source:HGNC Symbol;Acc:HGNC:8980] | 10956 | -0.425 | -0.2054 | Yes |
| 38 | PIK3R1 | phosphoinositide-3-kinase regulatory subunit 1 [Source:HGNC Symbol;Acc:HGNC:8979] | 11099 | -0.443 | -0.1830 | Yes |
| 39 | IRS2 | insulin receptor substrate 2 [Source:HGNC Symbol;Acc:HGNC:6126] | 11390 | -0.485 | -0.1691 | Yes |
| 40 | IRS1 | insulin receptor substrate 1 [Source:HGNC Symbol;Acc:HGNC:6125] | 11841 | -0.557 | -0.1620 | Yes |
| 41 | MTOR | mechanistic target of rapamycin kinase [Source:HGNC Symbol;Acc:HGNC:3942] | 12047 | -0.605 | -0.1323 | Yes |
| 42 | SOCS3 | suppressor of cytokine signaling 3 [Source:HGNC Symbol;Acc:HGNC:19391] | 12453 | -0.725 | -0.1092 | Yes |
| 43 | SOCS1 | suppressor of cytokine signaling 1 [Source:HGNC Symbol;Acc:HGNC:19383] | 12664 | -0.836 | -0.0625 | Yes |
| 44 | INSR | insulin receptor [Source:HGNC Symbol;Acc:HGNC:6091] | 12856 | -1.071 | 0.0034 | Yes |
Table: GSEA details [plain text format]

  

Fig 2: KEGG\_TYPE\_II\_DIABETES\_MELLITUS      
 Blue-Pink O' Gram in the Space of the Analyzed GeneSet

  

Fig 3: KEGG\_TYPE\_II\_DIABETES\_MELLITUS: Random ES distribution      
 Gene set null distribution of ES for **KEGG\_TYPE\_II\_DIABETES\_MELLITUS**
